# Supplementary material for: Unveiling the Molecular Landscape of FOXA1 Mutant Prostate Cancer: Insights and Prospects for Targeted Therapeutic Strategies
Source: Int J Mol Sci. 2023 Oct 31;24(21):15823. doi: 10.3390/ijms242115823 (PMC10650174; doi:10.3390/ijms242115823)
Supplement: Supplementary file 1 [file ijms-24-15823-s001.zip › Supplementary Data_proofreading.pdf]

Ijms-2627865

Unveiling the Molecular Landscape of *FOXA1* Mutant Prostate Cancer: Insights and Prospects for Targeted Therapeutic Strategies

Supplementary Data

Supplementary Figure S1. (A) Kaplan-Meier analysis plots comparing Overall Survival and Disease Free Months between *FOXA1* mutant and control groups. (B) A lollipop plot presenting the location of mutations and mutation types of *FOXA1* in *FOXA1* mutant prostate cancer. The plot was obtained from cBioportal.

A)

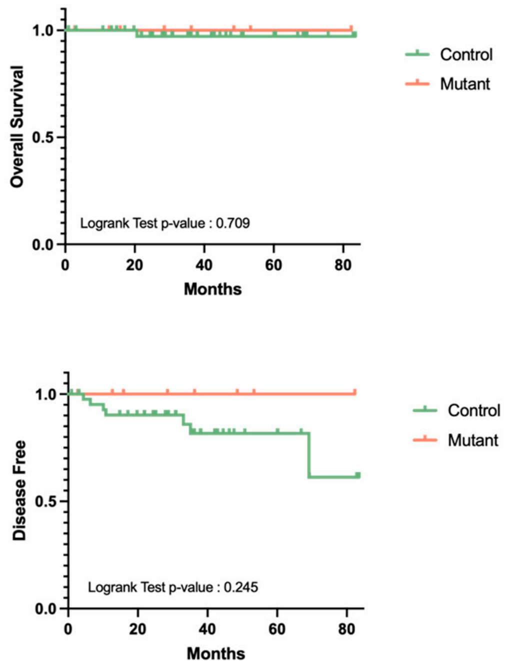

B)

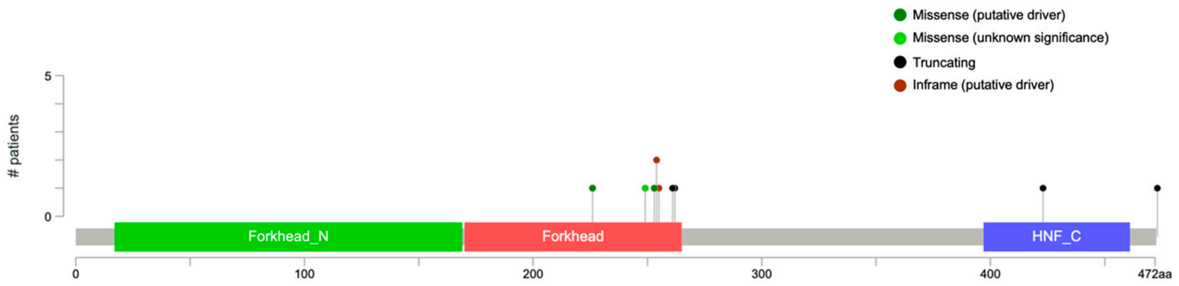

Supplementary Table S1. Detailed information of *FOXA1* mutations

Sample ID	Protein Change	Mutation Type	Variant Type
TCGA-YL-A9WI-01	D226N	Missense_Mutation	Single Nucleotide Polymorphism
TCGA-KK-A8IB-01	M253K	Missense_Mutation	Single Nucleotide Polymorphism
TCGA-CH-5737-01	E255_N256del	In_Frame_Deletion	Deletion
TCGA-HC-7210-01	F254_G257delinsC	In_Frame_Deleletion	Deletion
TCGA-KK-A8IG-01	Q263Afs*60	Frame_Shift_Insertion	Insertion
TCGA-EJ-5494-01	*473Eext*34	Nonstop_Mutation	Single Nucleotide Polymorphism
TCGA-EJ-5494-01	A423Dfs*17	Frame_Shift_Deletion	Deletion

TCGA-J9-A8CP-01	R262Sfs*57	Frame_Shift_Deletion	Deletion
TCGA-HC-8265-01	D249V	Missense_Mutation	Single Nucleotide Polymorphism
TCGA-EJ-7789-01	F254V	Missense_Mutation	Single Nucleotide Polymorphism

A)

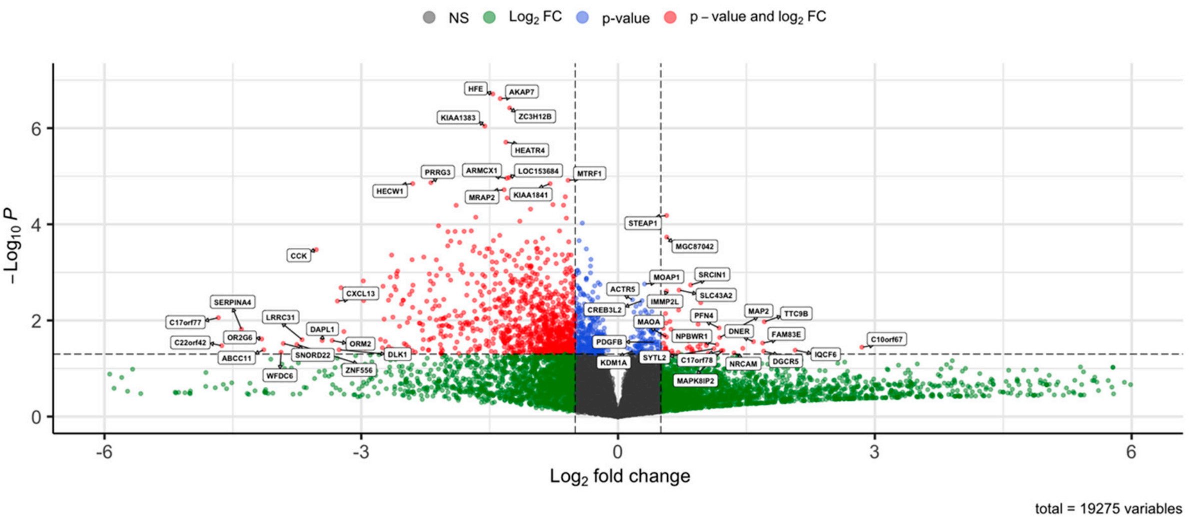

B)

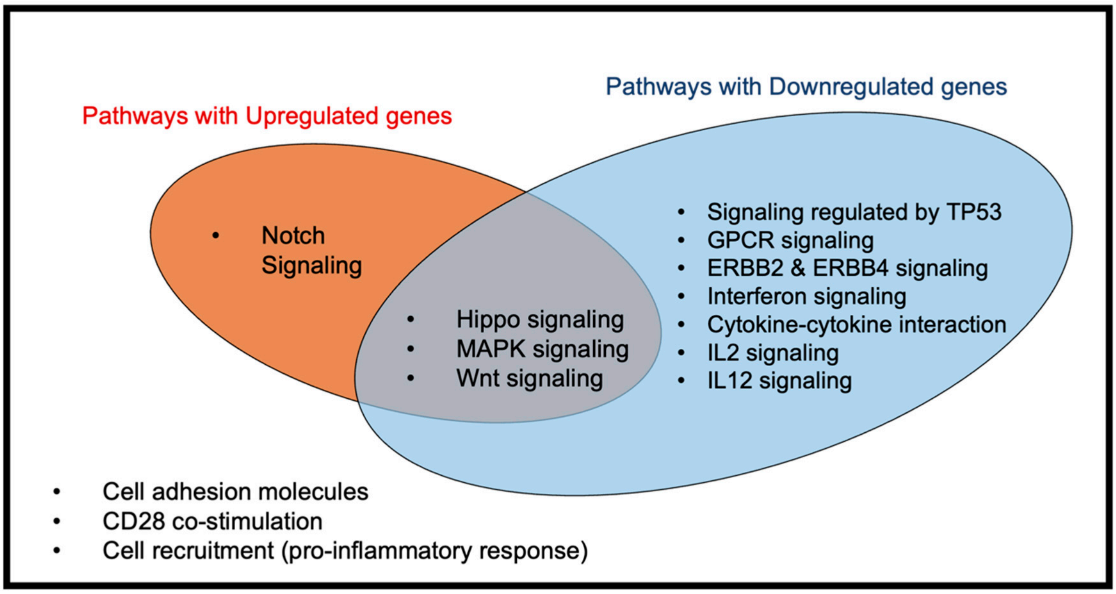

Supplementary Figure S2. (A) A volcano plot with Log2FoldChange showing the entire genes used in our analysis, except N/A or infinite values. Genes showing remarkable value of log2(Fold Change) or *p* value were marked with their genename. (Fold Change) = (mean expression in *FOXA1* mutation group)/(mean expression in control group). *NS = Not significant. (B) A Venn diagram displaying the results of pathway analysis with 168 upregulated genes and 1357 downregulated genes identified in *FOXA1* mutant group. Three pathways outside the Venn diagram only emerged from the pathway analysis involving all 1525 DEGs.

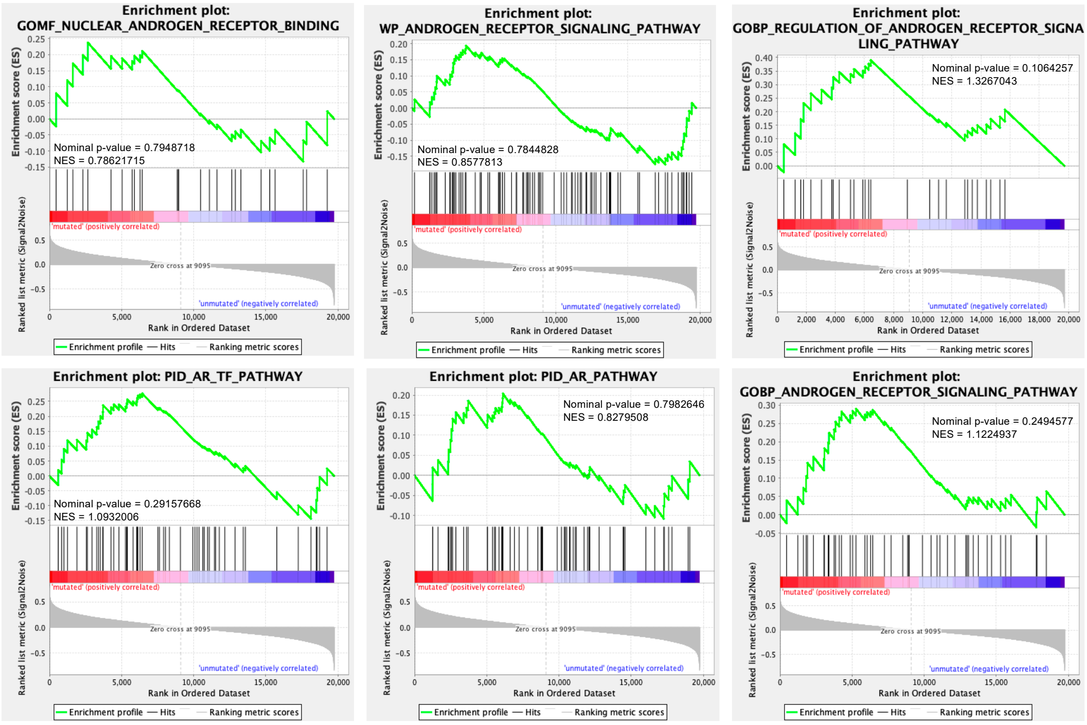

Supplementary Figure S3. GSEA analysis for AR signaling. GSEA analysis shows no significant difference in AR signaling in *FOXA1* mutant and control group.

*NES=Normalized Enrichment Score.

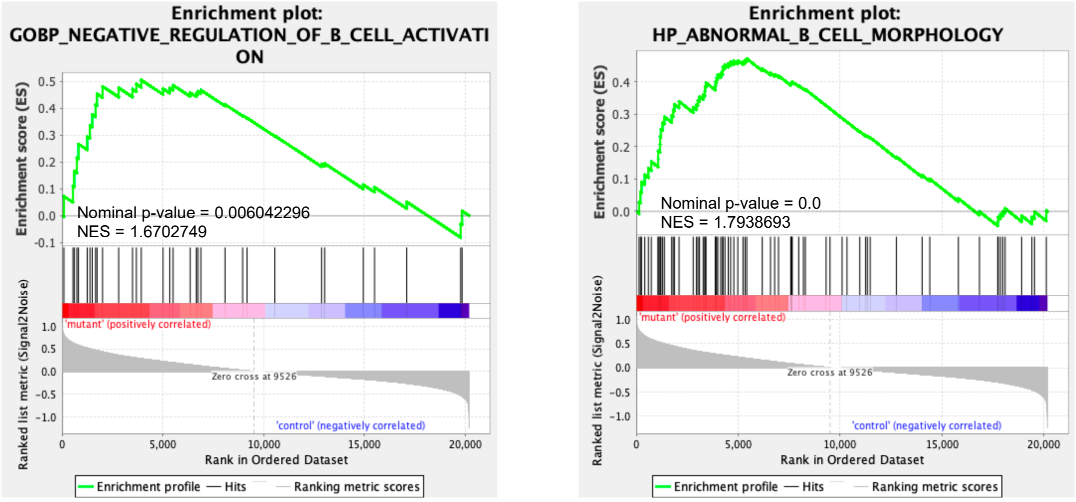

Supplementary Figure S4. GSEA analysis related to B cell. Negative regulation of B cell activation and abnormal B cell morphology were upregulated in *FOXA1* mutant group.

*NES=Normalized Enrichment Score.

Supplementary Table S2. *FOXA1* mutation-associated signaling pathways in TCGA and GEO datasets.

Pathways	*p*-value	
	**TCGA**	**GSE123619**
Hippo signaling pathway	0.007	0.018835332
ERBB signaling pathway	0.00229186	4.62334E-05
Interferon signaling	0.014055387	0.028836313
Notch signaling	0.01188312	0.046089423
Cell adhesion	0.04769012	0.000230778
Wnt signaling	0.00767968	0.045312137
Signaling regulated by TP53	0.00113	2.89E-07
MAPK signaling	0.03439121	7.65E-05
IL2 signaling	0.012385331	0.000129847

Supplementary Table S3. Monoamine neurotransmitter related pathways.

Pathways	*p*-value	
	**TCGA**	**GSE123619**
Dopamine beta-hydroxylase deficiency	0.000605401	0.038811814
Monoamine oxidase-a deficiency (MAO-A)	0.000605401	0.038811814
Serotonin	0.019527612	0.048104587

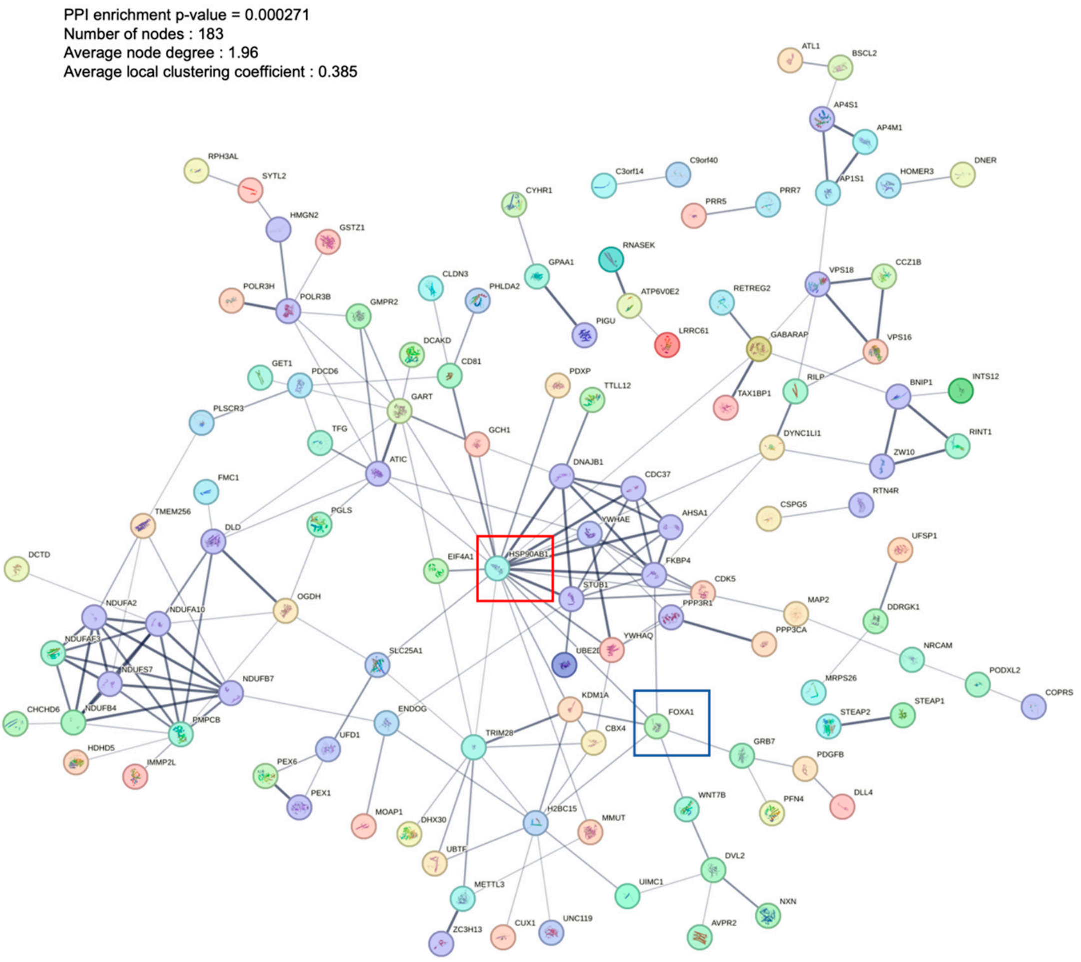

Supplementary Figure S5. Protein-protein interaction presented by STRING. The results displayed connected nodes representing proteins encoded by the 168 upregulated genes and other proteins mediating their interactions. The lines connecting proteins indicate both functional and physical protein associations, and the line thickness indicates the strength of data support. HSP90AB1 protein was marked by the red box and FOXA1 protein was marked by the blue box.
